# Analysis of the Radiological Changes of the Sinus Membrane Using Cone Beam Computed Tomography and Its Relationship with Dental Treatments. A Retrospective Study

**DOI:** 10.3390/biology11020165

**Published:** 2022-01-20

**Authors:** María Helena Rey-Martínez, Pedro Luis Ruiz-Sáenz, Natalia Martínez-Rodríguez, Cristina Barona-Dorado, Cristina Meniz-García, Jorge Cortés-Bretón Brinkmann, Juan Antonio Suárez-Quintanilla, José María Martínez-González

**Affiliations:** 1 Department of Otolaryngology, Central Hospital of the Red Cross of Madrid, 28003 Madrid, Spain; helena.rey@salud.madrid.org; 2 Department of Odontology, Central Hospital of the Red Cross of Madrid, 28003 Madrid, Spain; prsaenz@salud.madrid.org; 3 Department of Dental Clinical Specialties, Faculty of Dentistry, Complutense University of Madrid, 28040 Madrid, Spain; nataliamartinez@ucm.es (N.M.-R.); cbarona@ucm.es (C.B.-D.); jcortesb@ucm.es (J.C.-B.B.); jmargo@ucm.es (J.M.M.-G.); 4 Department of Morphological Sciences, School of Medicine, University Santiago de Compostela, 15782 Santiago de Compostela, Spain; juanantonisuarez.suarez@usc.es

**Keywords:** dental factors, iatrogenic factors, cone beam computed tomography (CBCT), sinus membrane thickening, odontogenic sinusitis

## Abstract

**Simple Summary:**

Changes in the sinus membrane, in the form of thickening or opacification, usually pose problems of differential diagnosis between rhinological and odontogenic causes, given the similarity in their clinical behaviour. The interrelation between tooth roots and the possibility of maxillary sinus involvement seems to be a key determinant. Moreover, the role played by iatrogenic factors, especially surgical interventions, such as dental extractions, or impacted teeth, as well as implant treatments, must be considered. The contribution of new imaging procedures, such as conventional computed tomography or cone beam computed tomography, has triggered an increase in the identification of dental aetiology as a cause of the unilateral opacification of the anterior paranasal sinuses with the predominant involvement of the maxillary sinus.

**Abstract:**

The aim of this study was to identify the most relevant dental factors and iatrogenic causes in the development of pathological changes to the sinus membrane and to analyse their possible influence on the development of odontogenic sinusitis. A descriptive, observational study was designed, with 276 patients who had been evaluated via cone beam computed tomography, analysing possible sinus thickening factors, such as apical infections, endodontic treatments, periodontitis, radicular cysts and impacted teeth, as well as iatrogenic factors caused by implant treatments or the development of oroantral communications produced during tooth extraction manoeuvres. Among the dental factors, periodontitis (47.1%), apical pathology (23.5%) and endodontic treatments (23.1%) were the predominant causes of sinus membrane thickening that most frequently produced an occupancy between 2 and 10 mm. Regarding the implant treatments, the placement of implants through the floor of the maxillary sinus was the main cause (9.8%), followed by sinus elevation techniques (6.2%). Dental extraction was the first cause of oroantral communication (5.0%), being the procedure that caused the greatest thickening of the sinus membrane. This study highlights the importance of dental treatments and iatrogenic factors in sinus pathology, and the need for diagnostic interrelations between the different specialists who address this pathology.

## 1. Introduction

Pathological changes in the maxillary sinus range from inflammatory processes to others of a cystic and tumoural nature. Among the former, maxillary sinusitis stands out, whose origin may be due to rhinological or odontogenic causes, and which is sometimes difficult to differentiate from the clinical point of view, so imaging diagnosis can play a relevant role [1].

The contribution of new imaging procedures has triggered an increase in the identification of dental aetiology as a cause of the unilateral opacification of the anterior paranasal sinuses with the predominant involvement of the maxillary sinus [2]. Matsumoto et al. [3] reviewed 190 CT scans of patients with unilateral sinus opacification, and found that more than 70% were attributed to odontogenic infection. Another radiographic study conducted by Bomeli et al. [4] found that the degree of opacification of the maxillary sinus was directly correlated with a concurrent dental source, finding that 79% of sinuses that were more than two-thirds opacified by liquid had an identifiable dental source.

The interrelation between tooth roots and the possibility of maxillary sinus involvement seems to be determinant and has been shown in different studies. Nascimento et al. [5] showed that the risk of a sinus disorder occurring when there is contact between the tooth and the maxillary sinus is increased by 2.77 times compared to cases without contact. De Lima et al. [6] observed that the smaller the distance that separates the roots with endodontic infection from the maxillary sinus, the greater the risk of chronic maxillary sinusitis. In contrast, a 2.5-fold decrease in risk was observed as the aforementioned distance increased. In a recent 2020 review by Peñarocha-Oltra et al. [7], the presence of periapical lesions was significantly associated with the thickening of the sinus membrane (OR 2.43) and maxillary odontogenic sinusitis (OR 1.77). Other authors have found that there are a variety of dental pathologies that can lead to odontogenic sinusitis, among which endodontic disease, periodontitis, oroantral communication or foreign bodies related to dental treatment stand out [8,9,10].

Despite this relatively high prevalence, some specialists tend to ignore odontogenic causes in the diagnosis and treatment of chronic sinusitis. Their description in the current guidelines for rhinosinusitis is scarce, and odontogenic causes are only briefly mentioned in the *European Position Paper on Rhinosinusitis and Nasal Polyposis* (EPOS), recently updated in 2020 [11].

The role played by implant treatments is, at present, another relevant aspect. Chen et al. [12] have shown a gradual increase in the incidence of sinusitis as a complication of dental implants. Among the different procedures, sinus elevations should be highlighted, as well as the penetration of the implants into the floor of the maxillary sinus and the placement of zygomatic implants.

Hernández Alfaro et al. [13] studied 474 sinus elevation procedures, observing a total of 104 membrane perforations. They concluded that small perforations usually heal spontaneously, and that the larger ones imply a risk of developing odontogenic sinusitis and implant failure. The perforation of the floor of the maxillary sinus by the most apical portion of the implants seems to be unrelated to the development of odontogenic sinusitis, as demonstrated by Jung et al. [14], who found no clinical signs of sinus infection. D’Agostino et al. [15] analysed the impact of treatments with zygomatic implants, observing that, among 26 maxillary sinuses operated with this procedure, 11.5% presented in the postoperative period a thickening of the sinus membrane.

The recognition of these dental and iatrogenic factors, mainly the implantological ones, should make the different specialists consider this alteration as a possible future source of clinical manifestations of such patients.

The objective of this work has been to assess the presence of changes in the sinus membrane by analysing the impact of dental pathology and iatrogenesis provoked during surgical and implant treatments.

## 2. Materials and Methods

### 2.1. Patient Selection

A descriptive, observational study was designed with patients belonging to the Oral and Maxillofacial Surgery Unit of the Faculty of Dentistry of the Complutense University of Madrid who had undergone complete maxillary cone beam computed tomography (CBCT) studies during the period from 2019 to 2020. A total of 276 patients were selected, in which the presence of sinus changes in one or both sinuses was observed. The study was carried out in accordance with the Declaration of Helsinki, and the protocol was approved by the Ethics Committee of the San Carlos Clinical Hospital in Madrid (CI 21/172-E).

For the selection and collection of data from patients’ clinical histories, the following inclusion criteria were established: (1) patients older than 18 years; (2) availability of a complete CBCT of the maxilla with panoramic, axial and ortho-radial sections; (3) presence of thickening of the sinus membrane in one or both maxillary sinuses; (4) existence of endodontic treatments, periapical radiolucency, periodontitis, impacted teeth and radicular cysts; (5) history of implant treatments; (6) iatrogenic triggers, such as simple teeth extractions and root fragments or third molar displacements.

The following were excluded from the study: (1) patients with a medical history of chronic rhinosinusitis, with or without polyps; (2) topical nasal corticosteroid treatments; (3) a history of having been treated in otorhinolaryngology; (4) changes suspected relating to malignant neoplasia of the sinonasal cavity.

### 2.2. Evaluation of Radiological Data

The radiographic findings were evaluated by four highly experienced observers (M.H.R-M.; C.B-D.; C.M-G.; J.M.M-G.), analysing through the different sections of the CBCT alterations of the sinus membrane, using NNT View, version 10.0 software (Newtom/Cefla S.C., Verona, Italy) with ‘Expert’ settings and in ‘Image Center’ application mode. In order to avoid bias in the measurements, fifteen CBCTs were evaluated by the observers; in cases with contrasting evaluations, we determined that at least three of them should coincide with their determinations to establish a concordance, with an intraclass correlation coefficient (ICC) of >0.9 between observers.

Regarding dental factors, the proximity of the root of the affected teeth to the sinus floor was measured as the shortest distance between the apex of the dental root and the floor of the maxillary sinus. Following the classification modified by De Lima et al. [6], the teeth were grouped into 5 types according to their relationship with the floor of the maxillary sinus: (0) the root apex is within the maxillary sinus; (1) the root apex protrudes towards the maxillary sinus; (2) the root apex is in contact with the floor of the maxillary sinus; (3) the root apex is between 0.1 and 1 mm below the floor of the maxillary sinus; (4) the root apex is more than 1 mm below the floor of the maxillary sinus.

The thickening of the mucosa was evaluated according to both dental and iatrogenic factors by measuring at the point of maximum thickness from the floor of the sinus, considering pathological inflammation of the sinus thickening of the mucosa greater than 2 mm according to the definition proposed by Maillet et al. [16]. The following parameters were established: (1) normal < 2 mm; (2) mild–moderate ≥ 2 mm; (3) severe > 10 mm; (4) total opacification.

### 2.3. Whyte and Boeddinghaus Aetiological Classification

Following the classification proposed by these authors [17], the findings were grouped into two categories: (1) dental pathology: periapical inflammatory pathology, due to a non-vital premolar or molar; periodontitis; and endodontic–periodontic pathology, which is the combination of the previous two. (2) Iatrogenic causes: oroantral communication/fistula, post-extraction of a molar; sinus elevation procedure to increase bone height for implant placement; and foreign bodies (root displacements, dental restorations and root canal fillings).

### 2.4. Statistical Analysis

For the statistical analysis, the statistical programme IBM SPSS Statistics for Windows (Version 27.0, IBM Corp., Armonk, NY, USA) was used to perform a detailed description of the data with frequencies and percentages. The association between variables was contrasted using the chi-square test and the standardized residuals were corrected from the crosstab tables, establishing a statistical significance of *p* ≤ 0.05.

## 3. Results

A total of 276 patients with a reconstruction of both maxillary sinuses were analysed in this study. Of these, 166 were men and 110 were women, establishing an M/F ratio of 1/0.66. The mean age was 56.67 years ± 14.02, with an interval of 18 to 90 years.

### 3.1. Dental Factors

**Figure 1:**
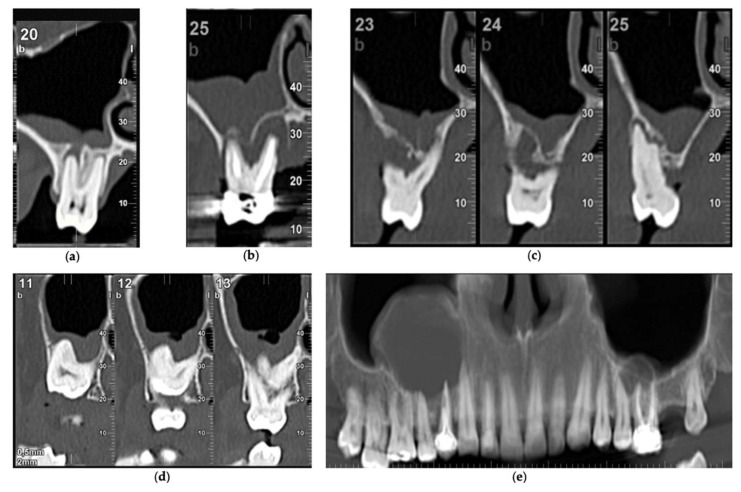
Dental factors triggering sinus thickening: (**a**) periapical pathology; (**b**) endodontic treatment; (**c**) periodontitis; (**d**) impacted third molar; (**e**) dental radicular cysts.

The presence of dental lesions was similarly observed in both maxillary sinuses (Table 1), and periodontitis was the main cause of sinus thickening, followed in order of frequency by endodontic treatments and periapical radiolucency (Figure 1).

### 3.2. Iatrogenic Factors

Iatrogenic factors were collected from the clinical histories of 69 patients of the total sample, and implant treatments represented 16.8%, compared with 8.3% for oroantral communications (Table 2).

**Figure 2:**
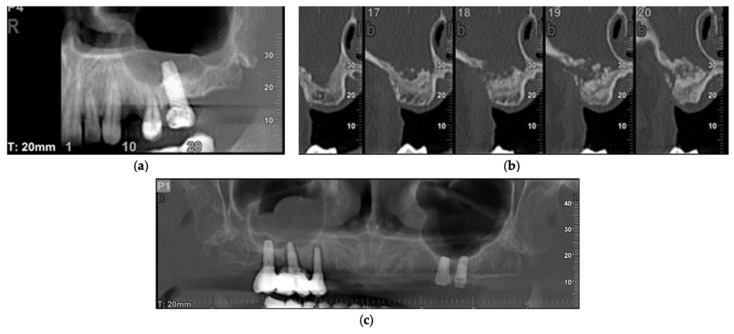
Implant treatments triggering sinus thickening: (**a**) implant exceeding the floor of the maxillary sinus; (**b**) sinus elevation failure; (**c**) peri-implantitis in the right maxillary sinus.

The placement of implants exceeding the floor of the maxillary sinus and causing thickening occurred in 9.8%, a failure in sinus elevation for implant placement was observed in 6.2% of cases, and peri-implantitis occurred in 0.8% (Figure 2).

**Figure 3:**
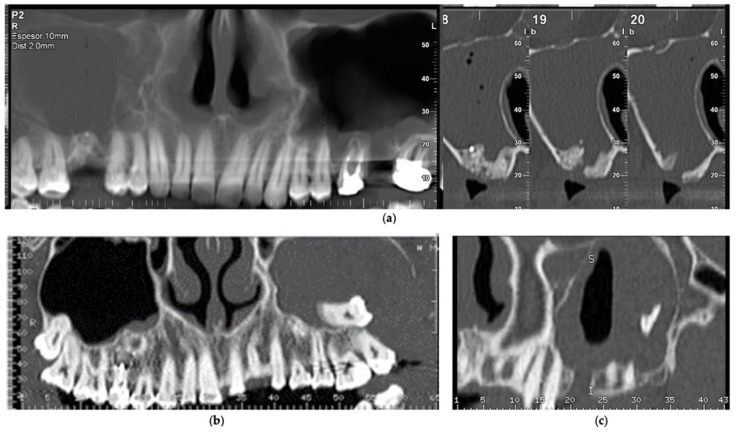
Oroantral communications triggering sinus thickening: (**a**) post-extraction communication of the upper right first molar; (**b**) displacement of the upper left third molar; (**c**) displacement of the tooth root to the left maxillary sinus.

Regarding oroantral communications, it was observed in the records that the main cause was tooth extraction, reaching a value of 5.0%, followed in decreasing order by root displacement with 1.8% and third molars with 1.5% (Figure 3).

### 3.3. Proximity of the Roots and Its Relationship with Dental Factors

In the analysis of the proximity of the roots, a homogeneous relationship was observed between both maxillary sinuses in the distribution of the five situations evaluated. The values (0), (1) and (2), which indicate a clear relationship of the roots with the maxillary sinus, were 68.2% for the right sinus and 74.9% for the left sinus (Table 3).

The relationship between the roots and the dental factors analysed highlights that on the right side, apical infection is mostly present in cases in which the dental roots protrude into the maxillary sinus. In periodontitis, these have been observed on both the right and left sides, preferably associated with dental roots that are in contact or at a distance of 1 mm. Odontogenic cysts were mainly related to the roots found inside the left maxillary sinus (Table 4).

### 3.4. Whyte and Boeddinghaus Classification

The classification proposed by these authors, which groups sinus pathology into six subtypes, has determined that dental pathology continues to be the first cause of change in the sinus membrane (subtypes 1, 2 and 3), with an overall mean value of 55.8%, with periodontitis being the main cause, representing 29.1% of cases. Iatrogenic causes (subtypes 4, 5 and 6) represented 17.55% of cases, with the foreign body subgroup being the main triggering factor, with an average value of 10.15% (Table 5).

### 3.5. Thickening of the Sinus Membrane and Its Interrelation with Dental Factors and Iatrogenic Causes

Considering that the pathological thickening of the sinus membrane is considered by Maillet et al. [16] for values ≥2 mm, the results are similar in both sinuses, with 55.0% for the right maxillary sinus and 59.61% for the left maxillary sinus.

The relationship between the thickening of the sinus membrane and the dental factors studied establishes that, in general, all of them can cause changes from minimal to most severe; however, apical infections are more likely to produce thickening between 2 and 10 mm, as occurs with periodontitis (Table 6).

When observing iatrogenic causes, oroantral communications produce greater thickening than implant treatments. Both irruption of the apical part of the implants in the maxillary sinus, as well as sinus elevation techniques when they fail, and peri-implantitis, present a greater probability of producing thicknesses between 2 and 10 mm. This thickening is much higher when oroantral communications occur after tooth extraction, the displacement of a root or the displacement of a third molar to the maxillary sinus (Table 7).

## 4. Discussion

The present research work addresses a widely studied topic, namely, dental factors and the thickening of the sinus membrane. However, it makes some significant contributions, such as providing a joint assessment of results by dentists and otolaryngologists, as well as the analysis of other factors that, in most publications, are not considered, such as odontogenic cysts. It has also highlighted that apical lesions are being overtaken by periodontitis, and that the rise of implant treatments is causing sinus problems that must be taken into account, such as peri-implantitis as a possible factor of thickening and contamination of the sinus membrane.

Changes in the sinus membrane in the form of thickening or opacification usually pose problems of differential diagnosis between rhinological and odontogenic causes, given the similarity in their clinical behaviour; therefore, radiographic studies play a fundamental role, with conventional computed tomography or CBCT being the current gold standard for accurate diagnosis [18].

Although radiographic findings, such as for periodontitis, oroantral fistula and periapical abscesses, are often present, radiologists commonly do not mention dental pathology as a source of sinusitis in their reports, as indicated by Vestin et al. [19]. Along the same lines, Allevi et al. [20], in a systematic review conducted in 2020, warned that not only radiologists, but also otorhinolaryngologists and dentists overlook the odontogenic aetiology as a cause of sinusitis, often due to an inadequate consensus on the pathological conditions to be considered.

The analysis of these odontogenic factors should be taken into account from the fifth decade of life, as aetiological causes of developing sinus pathologies become more prevalent from then. Matsumoto et al. [3], Pokorny et al. [18], and Lechien et al. [21] observed an average age between 40 and 50 years, figures somewhat lower than those observed in this study, in which the mean age reached values of 56.67 years ± 14.02 years. This agrees more with the recent review by Craig et al. [22], in which the mean age was 51.2 ± 3.9 years.

Gender predisposition was not uniform in the studies reviewed. The patients analysed in this study were mostly men, coinciding with other authors, such as Bajoria et al. [23] and Turfe et al. [24]. In contrast, Saibene et al. [25] and Arias-Irimia et al. [26] observed a greater involvement of women.

Periodontitis, endodontic treatments and periapical radiolucency were the most frequent findings observed in this study, the first being clearly superior. The scientific literature has attributed to periapical radiolucency and endodontic treatments a greater role in sinus changes; however, studies in the dental field are beginning to show that periodontitis can overtake these as an aetiological factor in changes to the sinus membrane. Thus, the role of periodontitis has been shown in different studies to be the main factor, with even higher figures than those found by us. Phothikhun et al. [27] showed that severe periodontal bone loss was significantly associated with the thickening of the membrane of the maxillary sinus for 42% of patients. Ren et al. [28], in a Chinese population, detected membrane thickening of ≥2 mm in 48.9% of patients with periodontitis. An explanation for these results is based on understanding how the prevalence of periodontitis increases with the age of the patient. As noted by Newsome et al. [29], in older patients, there is enough time for the loss of alveolar bone to occur and consequently to produce periodontitis.

One of the main challenges regarding these dental factors is the diagnostic criterion used to consider whether their impact on the sinus membrane can lead to the development of odontogenic sinusitis. Craig et al. [22], in a review conducted in 2021, noted that odontogenic sinusitis should be diagnosed based on the laterality of the disease, the symptoms, the findings of nasal endoscopy, the bacterial cultures of the sinuses, and the findings of the CT scan. They note that although opacification of the nasal sinuses or the thickening of the membrane on a CT scan may suggest sinusitis, these findings are nonspecific, and nasal endoscopy is more effective at confirming infectious sinusitis. However, if nasal endoscopy is normal or cannot be completed, patients could have been tentatively confirmed to have sinusitis based on suspicious symptoms or CT findings.

According to most publications, the laterality of sinusitis due to odontogenic causes is the most common (Table 8) [18,24,30,31,32,33,34,35,36,37,38,39,40]. However, we found in this study that bilateral changes were observed in 17.9% of patients, which is in line with the results obtained by Wang et al. [34] and Saibene et al. [25], who found bilateral involvement to be between 16 and 19% and to be 18.7%, respectively.

The symptomatology of odontogenic sinusitis should be assessed when two or more of the four key symptoms of sinusitis—nasal obstruction (congestion), nasal discharge (rhinorrhoea), facial pressure (pain or fullness), and anosmia or hyposmia—are present for at least 12 weeks [12,41]. The evaluation of these symptoms, to determine the presence of odontogenic sinusitis, can be performed, among others, with the Sino-Nasal Outcome Test 20 (SNOT-20), which consists of 20 indicators with scores using a Likert scale from 0 to 5, helping to assess the intensity of symptoms or their impact on quality of life [42].

Despite this, these clinical manifestations may not be present, or may be minimal. In the clinical histories of the patients included in this study, there were no signs or symptoms suggestive of sinusitis, with the exception of cases with oroantral communications, corroborating the observations made by Longhini and Ferguson [31], who only reported the presence of pain in 29% of cases of odontogenic sinusitis. Brook [43] and Tataryn et al. [44] agree that many patients may be asymptomatic due to the conserved permeability of the osteomeatal complex, which allows the release of pressure from the interior of the sinus. This is the reason why Whyte and Boeddinghaus [45] consider that unilateral opacification of the maxillary sinus is the most distinctive feature of odontogenic sinusitis without associated sinonasal symptoms in the absence of obstruction of the osteomeatal complex.

Establishing the level of opacification or thickening to diagnose odontogenic sinusitis can be complex, given the different opinions collected in the scientific literature. There are diverse estimations of the precise thickness of the sinus membrane at which it should be considered pathological. Thus, Liston et al. [46] and Papapanou et al. [47] considered there to be pathological thickening when there was an increase of >1 mm. For Maillet et al. [16] and Shanbhag et al. [48], this thickening would be pathological from values of >2 mm, and for Shahbazian et al. [49], it should be >3 mm. Where there does seem to be a consensus, at least in the dental literature, is considering a thickening of >10 mm to be severe [28,50,51]. In this research, the criteria proposed by Maillet et al. [16] were established, in which the thickening of the sinus membrane should be considered to be pathological when it reaches values of >2 mm, obtaining mean pathological values for both sinuses in 60.22% of the cases. These results are lower than those found by Nascimento et al. [5], who observed thickening of >2 mm in 86.9% of the cases, and higher than those observed in 2019 by Askoy et al. [52], who found a value of 42.1%.

The response of the sinus membrane to periapical pathology, endodontic treatments and periodontitis can be diverse, although apical infections, and especially periodontitis, play a greater role in the development of sinus thickening, in line with the article published in 2021 by Huang et al. [53], who found a significant association with bone loss and periapical radiolucency. The anatomical condition of the interrelation of the roots with the floor of the maxillary sinus seems to be a key element and has been mentioned by different authors, who report a significant association between the proximity of the diseased tooth roots to the sinus and the prevalence of disease of the sinus [5,54,55,56]. In the present study, this close relationship was observed in more than 70% of patients in both maxillary sinuses. This result is very similar to that observed by Bajoria et al. [23], who analysed 1000 maxillary sinuses, finding that in 74.9% of cases, the apex of any of the teeth touched the floor of the sinus, establishing that the incidence of thickening of the sinus membrane was higher in cases in which the tip of the root exceeded and protruded through the floor of the maxillary sinus.

This finding itself does not seem sufficient to enable the diagnosis of odontogenic sinusitis, given that it is necessary to evaluate the presence of signs and symptoms, as well as to extend the study of CBCT to other paranasal sinuses.

Another relevant aspect is the interrelation between odontogenic sinusitis and iatrogenic factors, whether relating ot surgical interventions, such as dental extractions, or impacted teeth, as well as implant treatments. Authors such as Akhlaghi et al. [57], in their systematic review, and Franco-Carro et al. [58], in a meta-analytic study, agree that the most common iatrogenic cause of odontogenic sinusitis is tooth extraction. Its frequency is estimated to be between 0.31% and 4.7%, and it usually occurs after the extraction of an upper molar or premolar, or by the displacement of the tooth root or of a third upper molar [59,60,61]. In these cases, the diagnosis of sinusitis is more accurate because sinus occupation is accompanied by clinical manifestations, as could be observed in the clinical histories of the patients, with pain and the sensation of fluid passing to the nose being the predominant symptoms.

The role that implant treatments currently play is undergoing a progressive growth due to the increase in population demand and, according to Chen et al. [12] and Kim et al. [62], implant treatments should be considered to be the main cause of odontogenic sinusitis. In these cases, it is convenient to differentiate between the cases in which the implants pierce the floor of the maxillary sinus in their apical zone and those in which a direct approach is performed for the increase in bone volume, such as sinus elevations, and evolving complications in the form of peri-implantitis. Of the 27 observed cases of perforated implants, the thickening occurred mostly in the 2–10 mm subtype, highlighting that none of the patients presented symptoms. Thus, this thickening should not justify the diagnosis of sinusitis. In the same vein, Jung et al. [14], who evaluated a group of patients in whom the implants had penetrated more than 4 mm in the maxillary sinus, found no clinical signs of sinusitis after a follow-up of ten months.

Sinus elevations are a different kind of situation, in which failure leads to a loss of regenerative material, as well as an almost complete opacification of the maxillary sinus. Moreover, failure is accompanied by clinical symptoms that identify the picture of sinusitis. For authors such as Mahesh et al. [63], Chiapasco et al. [64], and Saibene et al. [65], these interventions have become a routine procedure, meaning that more complications arise from their use when the integrity of the sinus membrane is damaged and the graft material migrates into the cavity, favouring bacterial contamination and subsequent sinus infection. Its frequency is increasing, as shown by the studies conducted by Manor et al. [66] in 2010, or Chirila et al. [67] in 2016, who established a frequency below 5%, to the most current frequency, as published in 2020 by Molteni et al. [68], where sinus elevations led to sinusitis in 10% of cases.

Although we have not found studies on the impact of peri-implantitis in the maxillary sinus, it can be assumed that the increase in this pathology may be an important factor of sinus contamination and, in advanced cases and as a consequence of the loss of the implant, establish oroantral communication with the consequent development of odontogenic sinusitis.

With regard to the Whyte and Boeddinghaus classification, we believe that although this can be used to compare with other studies, it should be modified in some respects. Regarding dental pathology, the impacted teeth and odontogenic cysts are not considered, entities that we have been able to verify that cause sinus changes. Similarly, and regarding iatrogenic factors, peri-implantitis should be incorporated.

Finally, although the affectation is predominantly in the maxillary sinus, we consider that one of the limitations of this research and, in general, of the CBCT for dental purposes (which usually provide panoramic, axial and orthoradial images), is the non-extension of the study to other paranasal sinuses and the osteomeatal complex. The measurements of the latter would allow researchers and clinicians to determine if the thickening of the mucosa is due to dental or multifactorial causes. In the same way, in future studies it would be advisable to carry out control groups and use the Lund–Mackay classification. This allows researchers and clinicians to evaluate each of the affected sinuses according to the following: (0) without opacification, (1) partial opacification and (2) total opacification, assessing the severity of the affectation as normal = 0, mild = 1–3, moderate = 4–10 and severe >10. In this way, it could be determined if dental factors, and especially iatrogenic factors, could be triggers for the involvement of other sinuses [69].

## 5. Conclusions

The thickening of the sinus membrane caused by dental factors is a common finding in CBCT studies, with periodontitis and apical pathology being the most common causes. Therefore, the evolution of this thickening will depend on the dental treatment of these affections, recommending the clinical and radiological follow-up of these patients.

On the contrary, iatrogenic factors, such as tooth extractions, still are the main causes for the development of orosinusal communication associated with odontogenic sinusitis. In addition, the increase in the number of implant treatments must be considered, in which the failure in sinus lift techniques is the main factor causing sinusitis. In these cases, the prognosis will depend on the surgical treatment carried out, either by closing the communication or removing the grafted material, given the essential need for CBCT to evaluate the changes in the sinus membrane.

## Figures and Tables

**Table 1:** Distribution of dental factors found in the right and left maxillary sinus by number of cases and percentages.

Dental Factors	RMS		LMS		Both		Total	
	No. Cases	%	No. Cases	%	No. Cases	%	No. Cases	%
Periapical pathology	31	11.2	28	10.1	6	2.1	65	23.5
Endodontic treatments	32	11.5	28	10.1	7	2.5	64	23.1
Periodontitis	49	17.7	50	18.1	31	11.2	130	47.1
Impacted teeth	10	3.6	8	2.8	5	1.8	23	8.3
Radicular cysts	3	1.1	3	1.1	1	0.3	7	2.5

RMS, right maxillary sinus; LMS, left maxillary sinus.

**Table 2:** Distribution of implant treatments and of the causes of oroantral communications by number of cases and percentages.

	RMS		LMS		Total	
**Implant treatments**	No. cases	%	No. cases	%	No. cases	%
Implants	14	5.1	13	4.7	27	9.8
Sinus elevations	6	2.2	11	4.0	17	6.2
Peri-implantitis	1	0.4	1	0.4	2	0.8
**Oroantral communications**	No. cases	%	No. cases	%	No. cases	%
Extractions	9	3.2	5	1.8	14	5.0
Root displacements	1	0.4	4	1.4	5	1.8
Third molar displacements	1	0.4	3	1.1	4	1.5

RMS, right maxillary sinus; LMS, left maxillary sinus.

**Table 3:** Distribution of the different relationship situations between the dental roots and the maxillary sinus by number of cases and percentages.

Proximity of the Roots	RMS		LMS	
	No. Cases	%	No. Cases	%
(0) Within	30	10.9	34	12.3
(1) Protruding	70	25.4	76	27.5
(2) Contact	88	31.9	97	35.1
(3) 0.1–1 mm	31	11.2	27	9.8
(4) >1 mm	57	20.7	42	15.2

RMS, right maxillary sinus; LMS, left maxillary sinus.

**Table 4:** Distribution, in percentages, of the different relationships between the dental roots and their statistical association with the dental factors.

Dental Factors	Proximity of Roots to RMS						Proximity of Roots to LMS					
	(0)	(1)	(2)	(3)	(4)	*p*	(0)	(1)	(2)	(3)	(4)	*p*
Periapical pathology	0.0	21.4	9.1	19.4	14.0	0.028	8.8	18.4	11.3	14.8	4.8	0.243
Endodontic treatment	26.7	14.3	13.6	16.1	7.0	0.172	2.9	18.4	12.4	11.1	11.9	0.261
Periodontitis	16.7	22.9	38.6	38.7	22.8	0.041	8.8	23.7	36.1	44.4	31.0	0.010
Radicular cysts	3.3	2.9	0.0	3.2	0.0	0.338	8.8	1.3	0.0	0.0	0.0	0.004

RMS, right maxillary sinus; LMS, left maxillary sinus; (0) within; (1) protruding; (2) contact; (3) 0.1–1 mm; (4) >1 mm.

**Table 5:** Distribution according to the Whyte and Boeddinghaus classification by number of cases and percentages.

Whyte and Boeddinghaus Classification	RMS		LMS	
	No. Cases	%	No. Cases	%
Without pathology	72	26.1	73	26.4
1. Periapical pathology (non-vital tooth)	37	13.4	34	12.3
2. Periodontitis	80	28.9	81	29.3
3. Endo-periodontal pathology (1 + 2)	39	14.1	35	12.6
4. Post-exo communication/fistula	9	3.2	14	5.0
5. Sinus elevation	6	2.2	11	3.9
6. Foreign bodies	16	5.7	23	8.3

RMS, right maxillary sinus; LMS, left maxillary sinus

**Table 6:** Distribution, in percentages, of the thickening of the sinus membrane and its statistical association with dental factors.

Dental Factors	RMS Thickening					LMS Thickening				
	(1)	(2)	(3)	(4)	*p*	(1)	(2)	(3)	(4)	*p*
Periapical pathology	27.0	37.8	21.6	13.5	0.021	20.6	47.1	17.6	14.7	0.106
Endodontic treatment	41.0	33.3	5.1	20.5	0.111	34.3	42.9	17.1	5.7	0.540
Periodontitis	31.3	47.5	12.5	8.8	0.014	27.2	44.4	16.0	12.3	0.054
Radicular cysts	50.0	0.0	25.0	25.0	0.349	0.0	50.0	25.0	25.0	0.361

RMS, right maxillary sinus; LMS, left maxillary sinus; (1) <2 mm; (2) 2–10 mm; (3) >10 mm; (4) total opacification.

**Table 7:** Distribution, in percentages, of the thickening of the sinus membrane and its statistical association with iatrogenic causes.

Iatrogenic Factors	RMS Thickening					LMS Thickening				
**Implant treatments**	(1)	(2)	(3)	(4)	*p*	(1)	(2)	(3)	(4)	*p*
Implants	28.6	64.3	7.1	0.0	0.110	23.1	69.2	0.0	7.7	0.111
Sinus elevation	0.0	50.0	0.0	50.0	0.004	9.1	63.6	0.0	27.3	0.026
Peri-implantitis	0.0	100.0	0.0	0.0	0.001	0.0	100.0	0.0	0.0	0.001
**Oroantral communications**	(1)	(2)	(3)	(4)	*p*	(1)	(2)	(3)	(4)	*p*
Exodontia	0.0	42.9	0.0	57.1	0.014	0.0	40.0	40.0	20.0	0.001
Root displacements	0.0	100.0	0.0	0.0	0.014	0.0	25.0	50.0	25.0	0.001
Third molar displacements	0.0	100.0	0.0	0.0	0.014	0.0	33.3	0.0	66.7	0.001

RMS, right maxillary sinus; LMS, left maxillary sinus; (1) <2 mm; (2) 2–10 mm; (3) >10 mm; (4) total opacification.

**Table 8:** Different studies have shown the mean age, gender, unilaterality and triggering factors of odontogenic sinusitis.

Authors	Study Design	No. Cases	Average Age	Gender	Unilateral	Dental Factors	Iatrogenic Factors
Lee and Lee [30], 2010	Retrospective	27	43	Male: 56% Female: 44%	Almost all	33%	67%
Longhini and Ferguson [31], 2011	Retrospective	21	53	Male: 48% Female: 52%	57%	100%	
Hoskison et al. [32], 2012	Retrospective	26	46	Male: 65% Female: 35%	Not reported	73%	27%
Pokorny and Tataryn [18], 2013	Retrospective	31	48	Male: 35% Female: 65%	94%	100%	
Crovetto-Martínez et al. [33], 2014	Retrospective	55	48	Male: 60% Female: 40%	100%	69%	31%
Wang et al. [34], 2015	Retrospective	55	55	Male: 40% Female: 60%	84%	58%	42%
Troeltzsch et al. [35], 2015	Retrospective	130	53	Male: 59% Female: 41%	100%	28%	68%
Zirk et al. [36], 2017	Retrospective	121	57	Male: 44% Female: 56%	92%	34%	66%
Ungar et al. [37], 2018	Prospective	25	49	Male: 36% Female: 64%	Not reported	16%	84%
Costa et al. [38], 2019	Retrospective	98	52	Male: 51% Female: 49%	100%	39%	61%
Craig et al. [39], 2019	Prospective	37	53	Male: 65% Female: 35%	89%	68%	32%
Turfe et al. [24], 2019	Prospective	60	55	Male: 58% Female: 42%	100%	70%	30%
Safadi et al. [40], 2020	Prospective	45	58	Male: 51% Female: 49%	Not reported	38%	62%

## Data Availability

The databases used and/or analysed during the current study are available from the corresponding author upon reasonable request.
